# Dispersed Crude Oil Induces Dysbiosis in the Red Snapper *Lutjanus campechanus* External Microbiota

**DOI:** 10.1128/spectrum.00587-21

**Published:** 2022-01-26

**Authors:** Andrea M. Tarnecki, Christelle Miller, Tracy A. Sherwood, Robert J. Griffitt, Ryan W. Schloesser, Dana L. Wetzel

**Affiliations:** a Marine Immunology Program, Mote Marine Laboratory, Sarasota, Florida, United States; b Environmental Laboratory for Forensics, Mote Marine Laboratory, Sarasota, Florida, United States; c Division of Coastal Sciences, School of Ocean Science and Technology, University of Southern Mississippi, Ocean Springs, Mississippi, United States; d Fisheries Ecology & Enhancement, Mote Marine Laboratory, Sarasota, Florida, United States; University of Texas at San Antonio

**Keywords:** *Deepwater Horizon*, *Lutjanus campechanus*, dispersed oil, external mucosa, microbiota, red snapper

## Abstract

The fish external microbiota competitively excludes primary pathogens and prevents the proliferation of opportunists. A shift from healthy microbiota composition, known as dysbiosis, may be triggered by environmental stressors and increases host susceptibility to disease. The *Deepwater Horizon* (DWH) oil spill was a significant stressor event in the Gulf of Mexico. Despite anecdotal reports of skin lesions on fishes following the oil spill, little information is available on the impact of dispersed oil on the fish external microbiota. In this study, juvenile red snapper (Lutjanus campechanus) were exposed to a chemically enhanced water-accommodated fraction (CEWAF) of Corexit 9500/DWH oil (CEWAF) and/or the bacterial pathogen Vibrio anguillarum in treatments designed to detect changes in and recovery of the external microbiota. In fish chronically exposed to CEWAF, immunoglobulin M (IgM) expression significantly decreased between 2 and 4 weeks of exposure, coinciding with elevated liver total polycyclic aromatic hydrocarbons (PAHs). Dysbiosis was detected on fish chronically exposed to CEWAF compared to seawater controls, and addition of a pathogen challenge altered the final microbiota composition. Dysbiosis was prevented by returning fish to clean seawater for 21 days after 1 week of CEWAF exposure. Four fish exhibited lesions during the trial, all of which were exposed to CEWAF but not all of which were exposed to V. anguillarum. This study indicates that month-long exposure to dispersed oil leads to dysbiosis in the external microbiota. As the microbiota is vital to host health, these effects should be considered when determining the total impacts of pollutants in aquatic ecosystems.

**IMPORTANCE** Fish skin is an immunologically active tissue. It harbors a complex community of microorganisms vital to host homeostasis as, in healthy fish, they competitively exclude pathogens found in the surrounding aquatic environment. Crude oil exposure results in immunosuppression in marine animals, altering the relationship between the host and its microbial community. An alteration of the healthy microbiota, a condition known as dysbiosis, increases host susceptibility to pathogens. Despite reports of external lesions on fishes following the DWH oil spill and the importance of the external microbiota to fish health, there is little information on the effect of dispersed oil on the external microbiota of fishes. This research provides insight into the impact of a stressor event such as an oil spill on dysbiosis and enhances understanding of long-term sublethal effects of exposure to aid in regulatory decisions for protecting fish populations during recovery.

## INTRODUCTION

Fish skin is immunologically active tissue that harbors diverse microbial communities, known as the microbiota, which interact intimately with their host (1). The mucosal skin surface is a primary entry point for pathogens but contains a vast repertoire of concentrated immunological factors to prevent infection. These include proteolytic enzymes, antimicrobial peptides, carbohydrate-binding proteins (lectins), and immunoglobulins (1). This protective layer also provides a site for adhesion by microorganisms capable of avoiding these immune factors, some of which gain nutrients from metabolizing compounds present in the mucus (2). The microbiota typically comprises numerous mutualistic and commensal species that prevent attachment of and produce antimicrobials against pathogenic microbes and thus acts as an extension of the fish’s immune system.

When the host is healthy, the microbiota is diverse and dominated by mutualists and commensals that prevent infection from primary pathogens and keep their abundance low in the community (2). Opportunistic pathogens are also common in the healthy microbiota (2); however, opportunists may overcome an immunocompromised host during times of altered homeostasis, establish infection, and cause disease. Physical and environmental stressors, such as changes in water parameters (i.e., temperature, pH, salinity, and oxygen), altered diet, and exposure to pollutants, antibiotics, and pathogens, are associated with deviations from the normal, healthy structure of the microbiota, a condition known as dysbiosis (3–5). Dysbiosis is characterized primarily by decreased bacterial diversity and increased relative abundance of primary and opportunistic pathogens (3–7). The imbalance of these communities reduces the microbiota’s protective ability, increasing the host’s susceptibility to infectious disease (6, 8).

The 2010 *Deepwater Horizon* (DWH) oil spill released nearly 5 million barrels of oil into the Gulf of Mexico, containing 2.1 × 10^10^ g of polycyclic aromatic hydrocarbons (PAHs) (9), which are oil constituents that are toxic in most organisms (10). In an attempt to distribute the plume throughout the water column and reduce coastal impacts, 2.1 million gallons of Corexit dispersant were applied to the ocean surface and injected into the DWH wellhead plume (11). In the decade since the DWH spill, PAHs and dispersants have received significant attention as environmental stressors. The effect of these pollutants on the health of fishes has been the topic of numerous investigations, with studies identifying immunosuppression (12, 13), oxidative stress (14), tissue damage (15), microbiota changes (15), and increased susceptibility to pathogens (13).

Following the DWH spill, there was an increase in anecdotal reports of fish species exhibiting unusual external lesions and ulcers (16). The observations of potentially impacted red snapper (Lutjanus campechanus) became a focal point of controversy surrounding the DWH spill as various media outlets reported on the presence of lesions (17–19), and studies by Murawski et al. (16) found a lesion rate of approximately 3% on this species in the year following the spill. Red snapper support an economically significant fishery in the United States, whose commercial landings value has increased by 73% since 2009, reaching a value of $32.8 million in 2019 (20). In the Gulf of Mexico, 16.6 million pounds of red snapper were landed in 2019, and with >80% of this harvest from recreational fisheries (21), these reports of lesions were of concern for local fishermen. Researchers failed to reject the hypothesis that the DWH spill caused these lesions due to correlations between ulcers and PAH concentrations in the fish (16). Further supporting a possible link between skin lesions and PAHs, red drum (Sciaenops ocellatus) collected from oil-contaminated sites exhibited a 20% higher incidence of skin lesions than those from oil-free reference sites (22). Additionally, southern flounder (Paralichthys lethostigma) exposed to oil through sediments developed bloody skin lesions similar to those reported in wild fishes (13). Immunosuppression in exposed flounder led to increased colonization by and mortality from the marine bacterial pathogen Vibrio anguillarum (formerly Listonella anguillarum) during experimental challenges (13). A previous study exposing red snapper to oil and V. anguillarum did not report lesions after 14 days; however, studies in Pacific herring suggest that longer-term exposure (29 days) is needed in order to increase susceptibility to this pathogen (23).

V. anguillarum is the causative agent of vibriosis, a disease characterized by hemorrhagic septicemia in marine fishes worldwide (24, 25). Signs of vibriosis in fishes include development of skin lesions that may become bleeding, open sores (25), like those reported following the DWH spill; however, the causative agent of the lesions during the DWH spill has not been determined. The main infection route for V. anguillarum is through the skin, particularly through injuries or damaged mucosa (25). The bacterium is lethal at seawater concentrations of 10^4^ CFU/mL and mortality may occur as soon as 5 days following exposure (25). The pathogen is attracted to fish mucus via chemotaxis, harbors resistance to fish-produced antimicrobials in these protective layers, and remains adhered to fish during shedding of the mucosal layer (25). Its role as a mucosal pathogen that can cause lesions similar to those seen in the DWH spill has made it a common challenge pathogen in oil exposure studies (12, 13, 23).

Sublethal impacts of PAHs include alteration of development, behavior, and gene expression, leading to reduced reproductive success, mutations, tissue damage, and long-term mortality (26). To truly understand population-level impacts, which are vital to assessing the extent of damage caused by contaminant exposures such as that during the DWH oil spill, we must understand how animals adapt following exposure. Few studies have investigated the impact of oil exposure on the fish microbiota. Bayha et al. (13) and Brown-Peterson et al. (15) detected a significant effect of oil exposure on the gill and intestinal microbiota of southern flounder after exposures of 1 month and 7 days, respectively. However, neither documented the ability of the microbiota to recover following dysbiosis. Larsen et al. (27) did not observe any impact of oil exposure on wild Gulf killifish (Fundulus grandis) external (skin-associated) microbiota a year after the DWH spill, perhaps identifying adaptation to or rehabilitation from contact with oil. Thus, measuring nonexposure versus exposure alone does not consider the organism's ability to recover following a stressor event.

**FIG 1:**
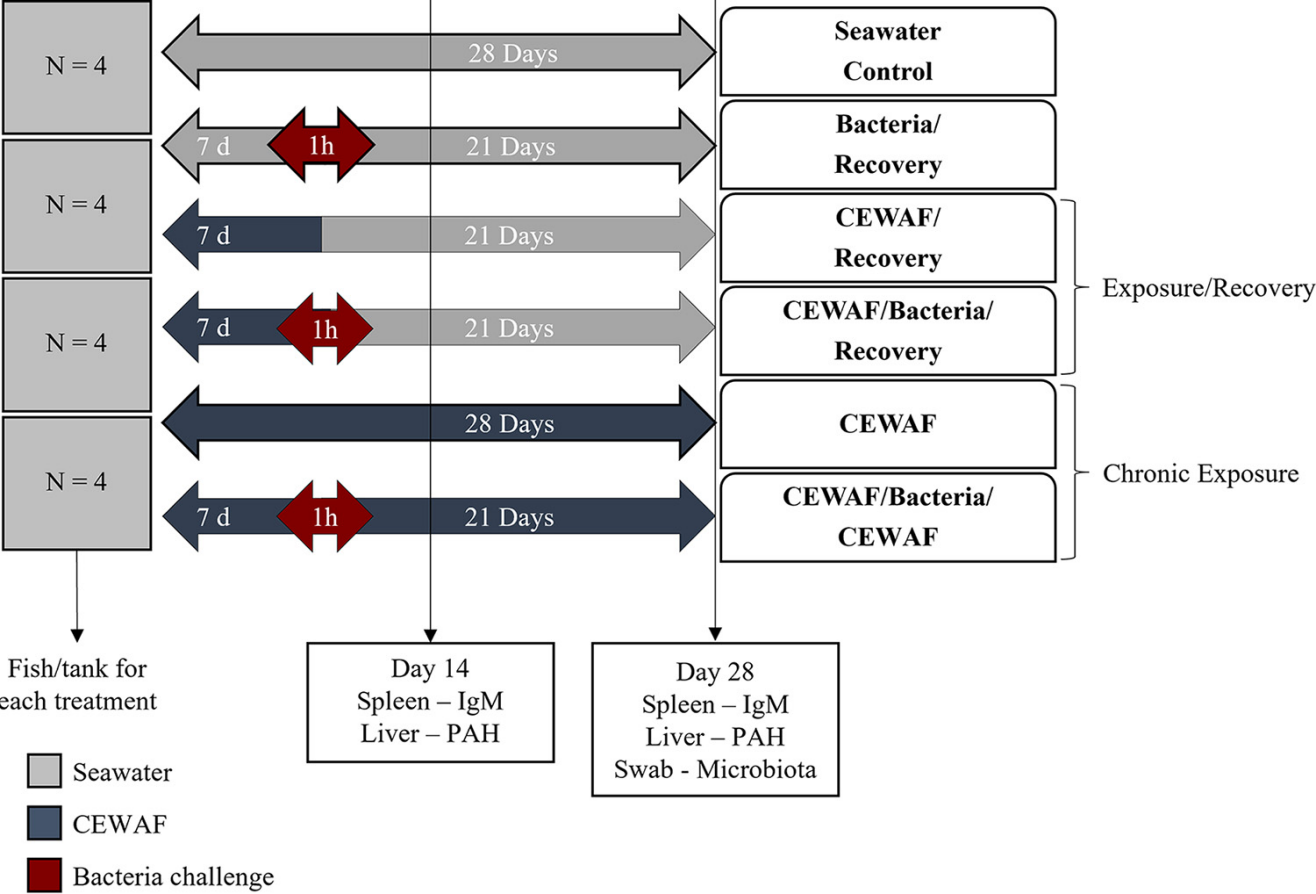
Experimental design and sampling points used in this study. CEWAF, chemically enhanced water-accommodated fraction; PAH, polycyclic aromatic hydrocarbon; IgM, immunoglobulin M.

The purpose of this study was to investigate the immunosuppressive effects of oil exposure on the external microbiota of red snapper. Microbial communities, PAH concentrations in fish liver, and immunoglobulin M (IgM) gene expression were monitored through a 4-week trial designed to determine the influence of exposure duration, recovery potential, and response to a combined exposure to dispersed DWH oil and the bacterial fish pathogen V. anguillarum. The experimental design is shown in Fig. 1. As the external microbiota is an extension of the host immune system, characterization of dysbiosis and the fish’s ability to recover from these altered communities provides insight into the chronic, sublethal effects of oil exposure on fish health at the individual and population levels.

## RESULTS

### Morphological observations.

There were no behavioral differences observed among treatments. There was no statistical difference in fish weights among treatments, nor was there a significant change in measurements between sampling days. Initial weights (average ± standard deviation [SD]) were 31.0 ± 15.9 g. Weights (average ± SD) were 37.8 ± 18.3 g and 33.8 ± 17.2 g for days 14 and 28, respectively. A few individuals exhibited skin lesions during sampling, including 3 from day 14 (one each from the chemically enhanced water-accommodated fraction of Corexit 9500/DWH oil [CEWAF]/Recovery, CEWAF/Bacteria/Recovery, and CEWAF treatment groups) and 1 at day 28 from CEWAF/Bacteria/Recovery (see Fig. S1 in the supplemental material).

### PAH concentrations in liver and CEWAF exposure solution.

The average ∑^48^ PAH concentration of the tank water for the 4-CEWAF treatments—two with 7 days only of CEWAF exposure and two with 28 days of CEWAF exposure—was 11.61 ± 9.37 μg/L. Livers were collected and analyzed from each of the exposure treatments at day 14 and again at day 28 (Table 1). The Seawater Control and Bacteria/Recovery fish had similar ∑^48^ PAH background levels, with an average of 304.19 ng/g wet weight at day 14 and 235.16 ng/g wet weight at day 28 (Table 2). The two treatments with 7 days of CEWAF exposure (CEWAF/Recovery and CEWAF/Bacteria/Recovery) averaged ∑^48^ PAH liver concentrations of 1,239.58 ng/g wet weight at the 14-day sampling time and decreased to 888.50 ng/g wet weight after 14 more CEWAF-free days. For the CEWAF constant exposures (CEWAF/Bacteria/CEWAF and CEWAF), there was an average ∑^48^ PAH liver concentration at the 14-day sampling time of 1,571.66 ng/g wet weight, which increased 250% to 4,040.19 ng/g wet weight by 28 days. The lower-molecular-weight (LMW) PAHs (where LMW represents 2 or 3 aromatic rings) comprised an average of >98% of the ∑^48^ PAHs in the liver of the day 14 CEWAF exposures (CEWAF, CEWAF/Recovery, CEWAF/Bacteria/Recovery, and CEWAF/Bacteria/CEWAF) compared to the higher-molecular-weight (HMW) PAHs (where HMW represents 4 or more aromatic rings), which comprised <2% of the analyzed PAHs (see Table S1 in the supplemental material). After the 28-day exposure, the liver LMW PAHs declined slightly to an average of 96%, while the HMW PAHs increased to approximately 4% of the total concentration (see Table S2 in the supplemental material). PAH concentrations in Seawater Control and Bacteria/Recovery are likely attributed to ambient laboratory conditions and are trivial to the ∑^48^ PAH measured in the livers of fish exposed to CEWAF.

**TABLE 1:** Number of samples analyzed during this study

Treatment	Day 14		Day 28		
	∑^48^ PAH	IgM	∑^48^ PAH^*a*^	IgM	Microbiota
Seawater Control	6 (3 composites of 2)	4	6 (3 composites of 2)	4	4
Bacteria/Recovery	6 (3 composites of 2)	4	8 (4 composites of 2)	4	4
CEWAF/Recovery	6 (3 composites of 2)	4	6 (3 composites of 2)	4	4
CEWAF/Bacteria/Recovery	6 (3 composites of 2)	4	8 (4 composites of 2)	4	4
CEWAF	6 (3 composites of 2)	4	8 (4 composites of 2)	4	4
CEWAF/Bacteria/CEWAF	6 (3 composites of 2)	4	10 (5 composites of 2)	4	4

a The number of targeted fish sampled for ∑^48^ PAH on each day was 6 per treatment, with a total fish load per treatment of 16 fish, allowing for potential mortalities. At day 28, all remaining fish were sampled.

**TABLE 2:** Liver PAH and IgM measurements in this study^*a*^

Treatment	Liver ∑^48^ PAH (ng/g)		IgM [log_2_(2^−ΔΔ^*^CT^*)]	
	Day 14	Day 28	Day 14	Day 28
Seawater Control	115.16 ± 80.18	81.84 ± 30.28	0.00 ± 1.97	0.00 ± 1.87
Bacteria/Recovery	571.96 ± 213.21	439.70 ± 42.51	0.04 ± 0.94	1.20 ± 0.84
CEWAF/Recovery	1,112.91 ± 422.41	551.57 ± 205.57	0.98 ± 1.58	0.62 ± 1.73
CEWAF/Bacteria/Recovery	1,366.25 ± 539.07	1,225.43 ± 991.87	0.70 ± 0.94	1.23 ± 1.45
CEWAF	1,406.19 ± 610.39	3,937.09 ± 2,164.88	2.28 ± 1.67	−1.04 ± 0.68
CEWAF/Bacteria/CEWAF	1,737.12 ± 646.90	4,122.66 ± 736.29	0.11 ± 1.01	0.28 ± 0.56

a The values shown are the average ± standard deviation.

### Chronic CEWAF alters IgM expression.

There was a significant interaction between treatment and sampling day (*P* < 0.05) for IgM expression in the liver, which was driven by significantly lower IgM expression on day 28 than on day 14 in fish within the CEWAF treatment group, likely due primarily to IgM suppression associated with increased PAH concentrations in the liver. On day 14, observed differences in IgM expression were associated with dispersed oil exposure, with CEWAF having higher IgM expression than other treatments, but due to the high variability among individuals and limited samples available, this suppression was not statistically significant (Table 2). On day 28, fish within the CEWAF treatment demonstrated suppression of the IgM response compared to other treatments, but as with day 14 results, this suppression was not statistically significant. Although not statistically significant, there was a trend for the bacterial challenge to enhance IgM response at day 28. This apparent increase was diminished when fish remained in CEWAF following bacterial exposure (CEWAF/Bacteria/CEWAF).

### CEWAF exposure induces dysbiosis in the red snapper external microbiota.

**FIG 2:**
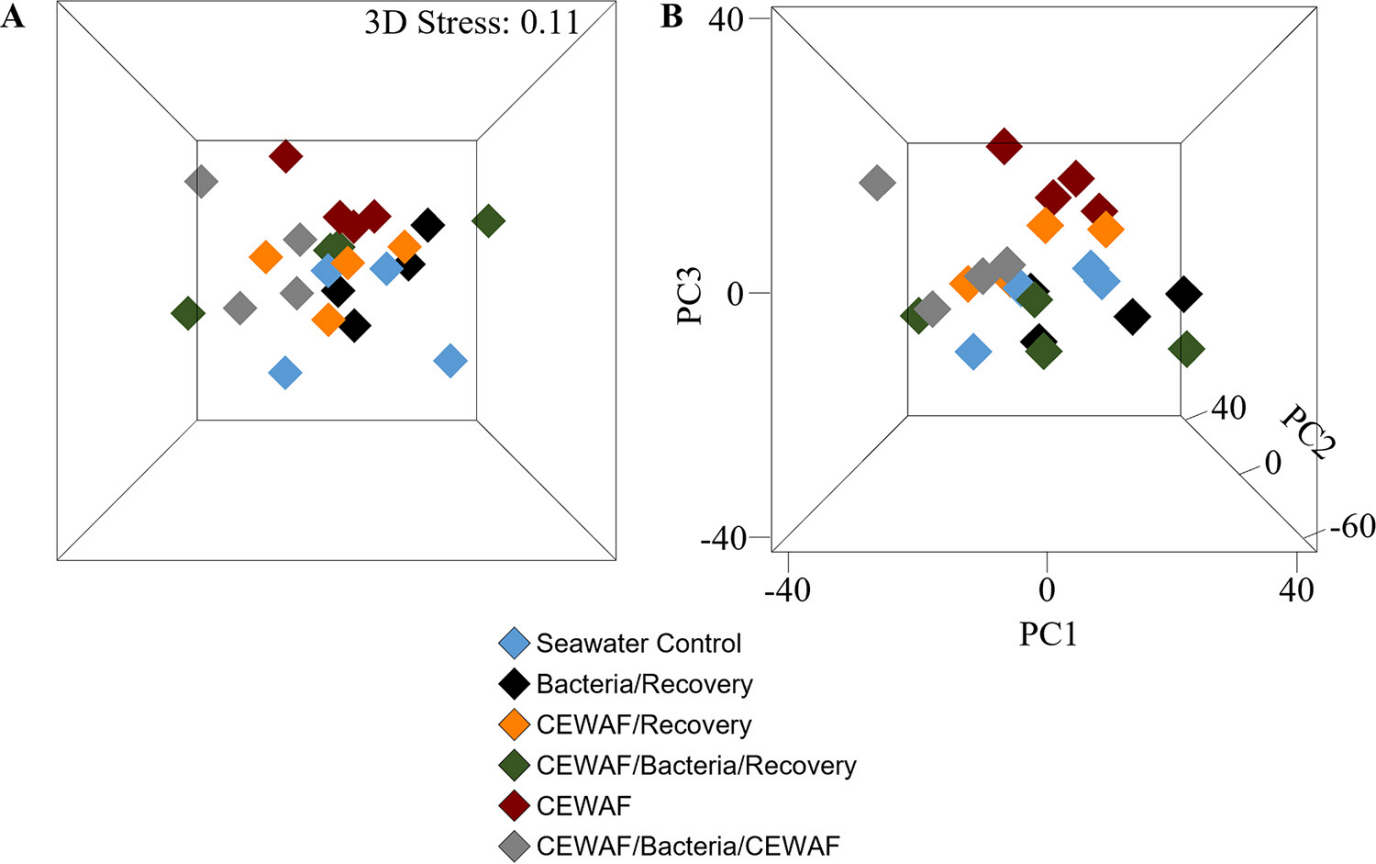
(A) Nonmetric multidimensional scaling (NMDS) and (B) principal-component analysis (PCA) analysis of red snapper external microbiota. *n* = 4 fish per treatment.

Good's coverage exceeded 99% in all samples, indicating thorough external microbiota sequencing (Table 3; see Fig. S2 in the supplemental material). There were no significant differences in any calculated alpha diversity measurements among treatments. Nonmetric multidimensional scaling (NMDS) and principal-component analysis (PCA) both suggested differences in microbiota structure between treatments (Fig. 2). These were confirmed statistically with permutational multivariate analysis of variance (PERMANOVA) (*P* = 0.001) and analysis of similarity (ANOSIM) (*P* = 0.001, *R* = 0.221). Both tests indicated the CEWAF treatment harbored statistically different microbiota than each of the other treatments. PERMANOVA detected significant differences between CEWAF/Bacteria/CEWAF and both Seawater Control and Bacteria/Recovery treatments, whereas these relationships were not significant using ANOSIM, with *P* values of 0.086 and 0.057, respectively. There were no other significant differences detected among overall community structures.

**TABLE 3:** Alpha diversity measures of the red snapper external microbiota^*a*^

Treatment	Good's coverage (%)	Richness	Shannon evenness index	Simpson index	Phylogenetic diversity
Seawater Control	99.6 ± 0.05	337 ± 60	0.66 ± 0.06	0.059 ± 0.032	27.6 ± 4.17
Bacteria/Recovery	99.6 ± 0.03	356 ± 54	0.68 ± 0.09	0.049 ± 0.037	29.6 ± 4.79
CEWAF/Recovery	99.6 ± 0.04	331 ± 43	0.66 ± 0.08	0.057 ± 0.050	27.8 ± 2.72
CEWAF/Bacteria/Recovery	99.6 ± 0.02	384 ± 59	0.59 ± 0.10	0.111 ± 0.095	31.6 ± 4.38
CEWAF	99.6 ± 0.07	341 ± 59	0.70 ± 0.05	0.038 ± 0.015	28.5 ± 2.70
CEWAF/Bacteria/CEWAF	99.6 ± 0.02	303 ± 65	0.68 ± 0.05	0.033 ± 0.014	26.3 ± 4.86

Avg	99.6 ± 0.05	342 ± 62	0.66 ± 0.08	0.058 ± 0.051	28.6 ± 3.96
*P* value	0.501	0.529	0.511	0.502	0.553

a The values shown are the average ± standard deviation (*n* = 4 per treatment).

**FIG 3:**
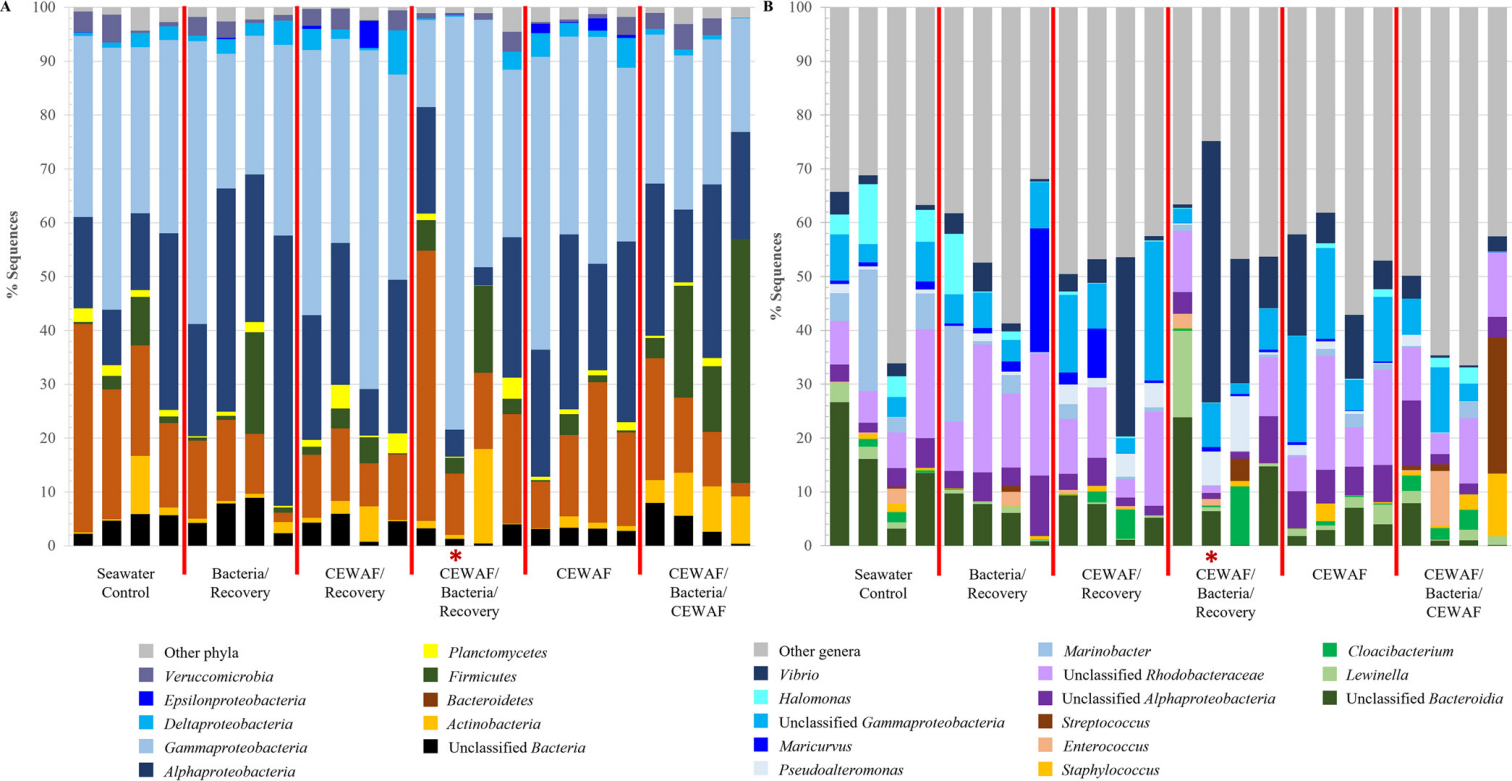
Taxonomic classification of red snapper external microbiota by individual and treatment. (A) Phylum-level classifications. (B) Genus-level classifications. The red asterisk indicates the individual with a lesion.

The red snapper external microbiota was dominated by members of the *Proteobacteria* and *Bacteroidetes*, with some individuals harboring relatively high abundances of *Firmicutes* (Fig. 3A). The *Gammaproteobacteria* composed over 75% of the microbiota of the lesioned individual, compared to an average of approximately 35% across all other individuals. Nearly 49% of sequences in the lesioned individual were ascribed to the genus *Vibrio* (Fig. 3B), with the next highest abundances of this genus found in an individual within CEWAF/Recovery (33%) followed by another individual within the CEWAF/Bacteria/Recovery treatment (23%).

**FIG 4:**
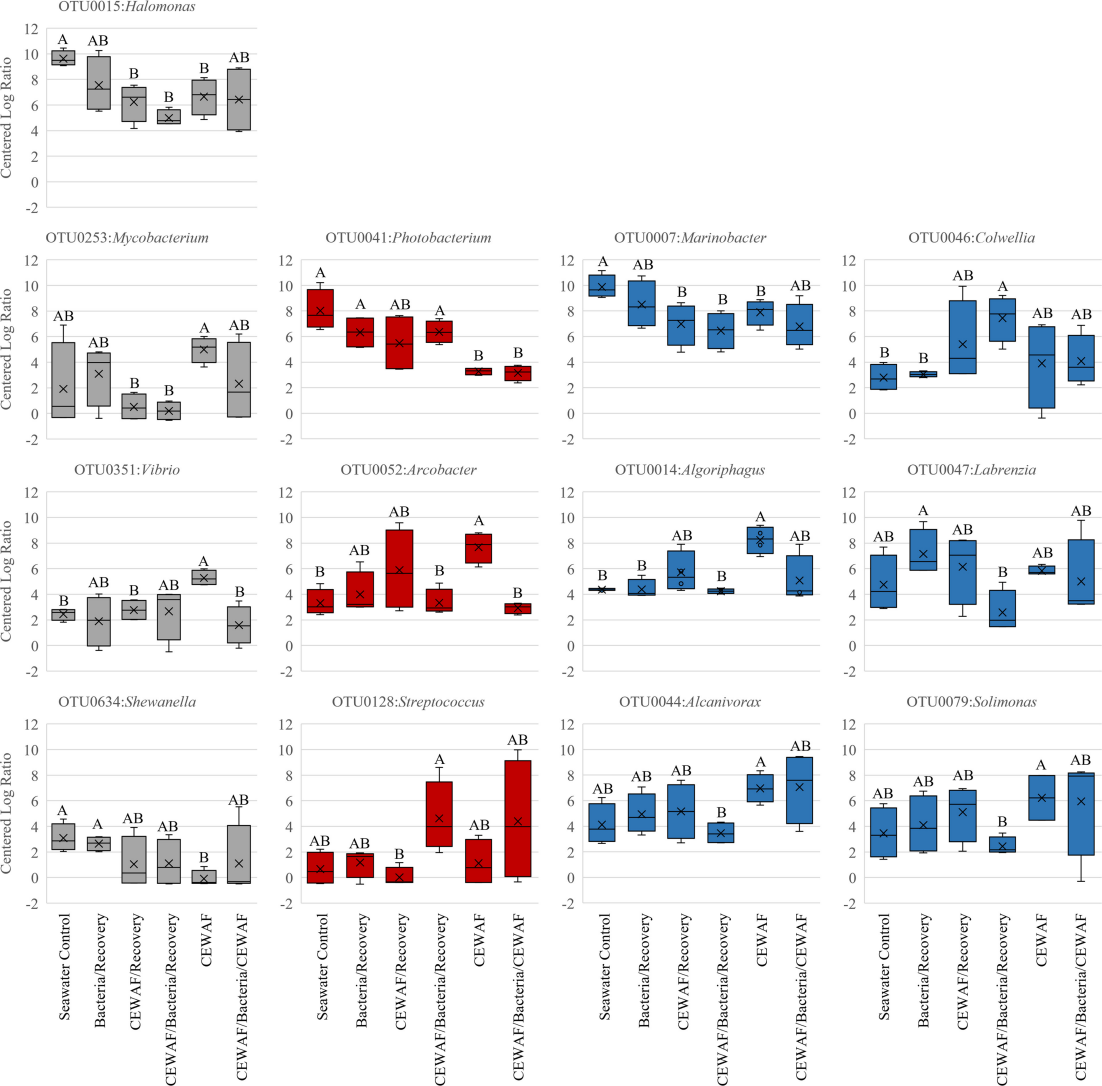
Differentially abundant OTUs among treatments. OTUs included in this figure were able to be classified to the genus level and contain members that have been identified as fish pathogens and/or hydrocarbon degraders. Gray indicates members that contain both fish pathogens and hydrocarbon degraders, red indicates members that contain fish pathogens only, and blue indicates members that contain hydrocarbon degraders only.

A total of 58 operational taxonomic units (OTUs) were determined to be differentially abundant between at least two treatments in both ALDEx2 and ANCOM (analysis of composition of microbes) analyses (see Table S3 in the supplemental material). OTUs that could be classified to the genus level and that contain members identified as hydrocarbon degraders (28, 29) and/or fish pathogens (24) are shown in Fig. 4. Within the potential fish pathogens, OTU0041, identified as *Photobacterium*, was on average more abundant in fish maintained in seawater at day 28 than in fish chronically exposed to CEWAF. OTU0052 *Arcobacter* was significantly higher in the CEWAF treatment than the Seawater Control, CEWAF/Bacteria/Recovery, and CEWAF/Bacteria/CEWAF treatments. OTU0128 Streptococcus was generally higher in fish exposed to both CEWAF and a bacterial challenge, with this difference only significant between CEWAF and CEWAF/Recovery. Six differentially abundant OTUs fell into taxa containing known hydrocarbon degraders. OTU0007 *Marinobacter* was significantly higher in Seawater Control than in the CEWAF/Recovery, CEWAF/Bacteria/Recovery, and CEWAF treatments. OTU0047 *Labrenzia* was more abundant in Bacteria/Recovery than in CEWAF/Bacteria/Recovery. OTU0014 *Algoriphagus* was significantly higher in CEWAF than Seawater Control, Bacteria/Recovery, and CEWAF/Bacteria/Recovery. Both OTU0044 *Alcanivorax* and OTU0079 *Solimonas* were on average more abundant in chronically exposed treatments but were significant only between CEWAF and CEWAF/Bacteria/Recovery. OTU0046 *Colwellia* was more abundant in Recovery treatments, but only significantly so when CEWAF/Bacteria/Recovery was compared to Seawater Control and Bacteria/Recovery. Four OTUs were identified as genera that contain members associated with fish disease and hydrocarbon degradation. Of these, OTU0015 *Halomonas* and OTU0634 *Shewanella* were generally more abundant in fish never exposed to CEWAF. For OTU0015, Seawater Control harbored significantly higher abundances than CEWAF and both Exposure/Recovery treatments, whereas OTU0634 was higher in Seawater Control and Bacteria/Recovery than CEWAF. OTU0253 Mycobacterium was significantly higher in CEWAF treatment than both Exposure/Recovery treatments. OTU0351 *Vibrio* was significantly higher in CEWAF than Seawater Control, CEWAF/Recovery, and CEWAF/Bacteria/CEWAF.

**FIG 5:**
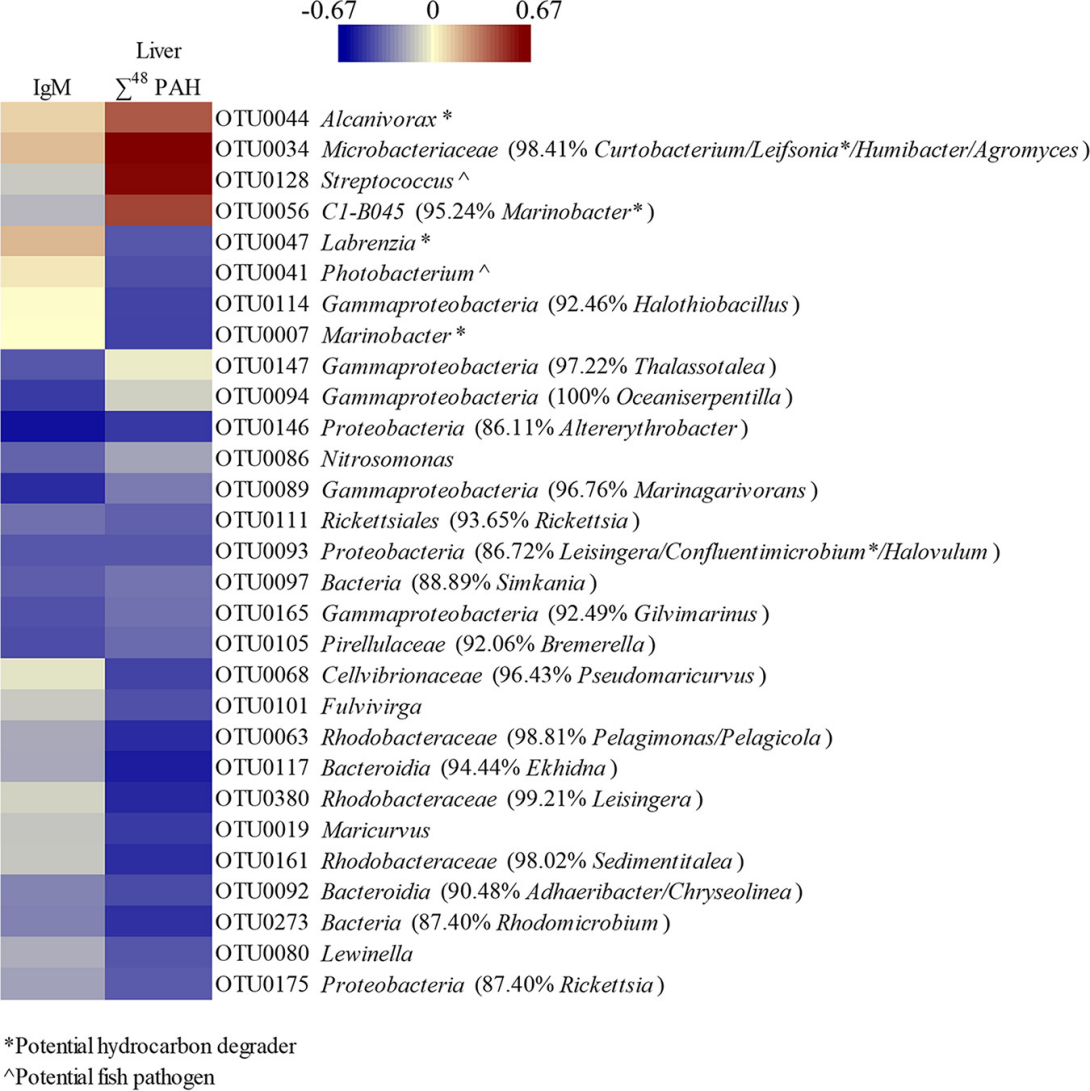
Correlations between OTUs and liver ∑^48^ PAH and IgM expression. Only OTUs with a correlation of ≥0.3 for at least one variable are included.

Differentially abundant OTUs were correlated with IgM and liver ∑^48^ PAH using partial least-squares (PLS) regression and visualized using clustered image maps (CIM) (see Fig. S3 in the supplemental material). Twenty-nine OTUs were correlated at 0.3 or greater with at least one variable (Fig. 5). The OTUs positively correlated with liver ∑^48^ PAH included OTU0044 *Alcanivorax*, OTU0034 *Microbacteriaceae*, OTU0128 Streptococcus, and OTU0056 C1-B045. The greatest negative correlations with liver ∑^48^ PAH include 3 OTUs identified as *Rhodobacteraceae* (OTU0063, OTU0380, and OTU0161), OTU0117 *Bacteroidia*, and OTU0273 *Bacteria*. The highest positive correlations with IgM included OTU0014 *Algoriphagus* and OTU0061 OM190, while the greatest negative correlations with IgM included OTU0146 *Proteobacteria* and OTU0089 *Gammaproteobacteria*.

## DISCUSSION

During this study, ∑^48^ PAH accumulated in the liver with exposure to CEWAF, which supports other studies indicating that PAHs accumulate in fatty, high-blood-flow tissues (26). The concentrations measured in this study, with an average of approximately 1,400 ng/g in the liver at day 14 of fish exposed to CEWAF, are within ranges reported in other oil exposure studies (14, 30) and wild fishes (31, 32). The higher concentrations (≈4,000 ng/g) detected in liver after a 28-day exposure to dispersed oil have been measured in wild fish livers (33, 34) and are thus not outside the potential concentrations detected in nature.

Some lab-based studies report a decrease in IgM expression following oil exposure (12, 13); however, these studies used crude oil or high-energy water-accommodated fractions (HEWAF) for exposures. Interestingly, Jones et al. (35), who exposed sheepshead minnows to HEWAF and CEWAF and measured gene expression using microarrays, found increased expression of genes associated with antibody-mediated immune response and disease resistance in CEWAF-exposed fishes versus HEWAF treatments, suggesting CEWAF leads to activation of the antibody response. Gulf killifish collected from natural environments showed increased expression of IgM heavy-chain genes in liver tissue when exposed to DWH versus control sites, indicating activation of the adaptive immune response (36). As samples were collected in late June, these fish may have been exposed to dispersant, which was applied starting in May following the spill (11). In our study, IgM expression was notably increased at day 14 for fish exposed to dispersed oil compared to day 28, when the antibody expression was reduced from chronic exposure. This contrasted with stable antibody expression in their clean seawater and pathogen-challenged counterparts. Although not statistically significant, a trend in IgM data suggests an increase in expression on day 28 in treatments where V. anguillarum-challenged individuals were maintained in clean seawater (Bacteria/Recovery, CEWAF/Bacteria/Recovery). However, fishes maintained in CEWAF following challenge (CEWAF/Bacteria/CEWAF) had reduced expression compared to these other treatments, indicating potential suppression of immune function in response to bacterial pathogens following chronic CEWAF exposure. Similarly, Bayha et al. (13) reported greater suppression of IgM in flounder challenged with oil and V. anguillarum than in fish exposed to oil alone. However, a similar study in red snapper exposed to crude oil and V. anguillarum did not exhibit these trends (12). Rodgers et al. (12) used HEWAF and a higher pathogen concentration (7.5 × 10^5^ CFU/mL), and concluded the study after 17 days, any of which may contribute to differences in immune response between our red snapper and the ones in that study. Overall, these results and those of other studies suggest differing effects on immune function when fish are exposed to crude oil versus CEWAF and point to potential immunosuppression and a decreased ability to launch an antibody response to pathogens in chronically exposed individuals.

Four fish developed lesions during the study, all exposed to CEWAF but not all exposed to V. anguillarum. This suggests that skin lesion development was driven more by the exposure to dispersed oil than to this bacterial pathogen. Microbiota data collected from the individual sampled at day 28 indicated that *Vibrio* is associated with lesion formation, as the external microbiota of this lesioned fish was composed of nearly 49% *Vibrio* sequences versus an average of 6% in nonlesioned fish. Unfortunately, *Vibrio* cannot be identified to the species level using 16S rRNA genes alone (37), so although one OTU was primarily responsible for the high relative abundance of the genus, we cannot determine which species it represents. Rodgers et al. (12) did not report lesions in red snapper exposed to HEWAF and V. anguillarum for 17 days, whereas Bayha et al. (13) observed hemorrhagic lesions in flounder within 2 days. Pacific herring were protected against V. anguillarum for up to 29 days following oil and pathogen exposure, but their susceptibility increased at that time (23). The variation in lesion appearance among these studies may be due to exposure material (crude oil versus oil and dispersant), exposure time, species-specific physiological responses, or life history, as red snapper are reef associated, flounder are benthic, and herring are pelagic. Other unmeasured parameters may also contribute to lesion formation, as research indicates metal exposure is associated with the appearance of lesions in wild fishes (38) and increased susceptibility to V. anguillarum (39).

Community-level analyses indicated that the external microbiota of chronically exposed red snapper (CEWAF and CEWAF/Bacteria/CEWAF) were significantly different from those of Seawater Control and Bacteria/Recovery fish, indicating that CEWAF exposure induces dysbiosis in the fish external microbiota. However, the bacterial challenge combined with chronic CEWAF exposure altered the external microbiota differently than exposure to CEWAF alone, as these two treatments harbored distinct communities from one another and 13 OTUs were differentially abundant between these two treatments. Fish exposed to CEWAF only maintained distinct microbiota compositions from those exposed to both Exposure/Recovery treatments, whereas the CEWAF/Bacteria/CEWAF group was not significantly different from either of these. We hypothesize these differences were due to a combination of pathogen challenge and physiological changes within the fish, as demonstrated by changes in IgM expression over time in CEWAF. As the cross talk between the fish immune system and mucosal microbiota results in alterations of the immune response by bacteria and vice versa, the changing IgM response may provide some insight into the continued dysbiosis detected in CEWAF-only individuals compared to their bacterium-challenged counterparts. The microbiota of fish within the Exposure/Recovery treatments were not statistically distinct from the Seawater Control or Bacteria/Recovery treatments, or each other, indicating that clean seawater for 21 days post-CEWAF exposure prevents dysbiosis in the fish microbiota.

Dysbiosis was also detected at the OTU level. Patterns of OTUs identified as *Halomonas* and *Marinobacter* were nearly identical across treatments, with Seawater Control fish containing higher abundances than those receiving the Recovery treatments and CEWAF only. An OTU classified as *Shewanella* was also higher in Seawater Control and Bacteria/Recovery than in CEWAF only. These genera have been reported previously in the external microbiota (7, 40–42) and may provide benefits such as stimulation of immunity (43–45), as well as increasing growth and nutritional conditions (46). These genera include known crude oil degraders (29); however, the OTUs were either negatively correlated or lacked correlation with liver PAH and may not be functioning primarily as crude oil degraders in this study.

Few OTUs were differentially abundant between Exposure/Recovery treatments compared to other treatments, supporting lack of dysbiosis with 3 weeks of clean seawater post-CEWAF exposure. One OTU identified as *Colwellia* was significantly more abundant in the CEWAF/Bacteria/Recovery treatment compared to Seawater Control and Bacteria/Recovery. This genus contains strains that are known crude oil degraders (29) and dominate microbial communities of diluted plumes (47), pointing to a role in later stages of degradation. In a mesocosm study, the genus *Colwellia* was dominant only in treatments that received dispersant, and it was hypothesized that these bacteria participate in the metabolism of the sulfur compounds resulting from dispersant use (48). The enrichment of *Colwellia* in CEWAF/Bacteria/Recovery occurred when petroleum hydrocarbons were no longer being added, and the genus was positively correlated with liver PAHs that remained in these fish following 3 weeks of recovery. Thus, the selection for this genus may reflect the natural hydrocarbon degradation process that has been detected in open water marine environments. Perhaps worth noting is one OTU identified as Streptococcus, which was significantly more abundant in CEWAF/Bacteria/Recovery than CEWAF/Recovery. This OTU also reached high abundances in some individuals within the CEWAF/Bacteria/CEWAF treatment. Streptococcus is generally reported in diseased fishes (49, 50), and in this study, it was strongly positively correlated with liver PAH concentrations.

Differential abundance analysis indicated a number of OTUs that were enriched in the CEWAF treatment. One OTU identified as *Algoriphagus*, a genus that contains species capable of degrading crude oil (29), was significantly more abundant in CEWAF fish compared to Seawater Control, Bacteria/Recovery, or CEWAF/Bacteria/Recovery fish. An OTU within the genus *Alcanivorax* was enriched in CEWAF versus CEWAF/Bacteria/Recovery. This genus is well known for its ability to degrade alkanes (29), is enriched in oil-contaminated environments (51, 52), and is more abundant in flounder exposed to oiled sediments (13, 15). The genus *Arcobacter* was also represented by an OTU that was significantly more abundant in CEWAF than in the Seawater Control, CEWAF/Bacteria/Recovery, and CEWAF/Bacteria/CEWAF groups. Members of this genus can cause disease in fishes (24) and were also enriched in flounder exposed to oiled sediments (15). Additionally, an OTU identified as Mycobacterium was enriched in CEWAF compared to either Exposure/Recovery treatment. The genus contains members capable of degrading phenanthrene hydrocarbons (29) and causing disease in fishes (24). A *Vibrio* OTU was significantly enriched in CEWAF compared to Seawater Control and CEWAF/Recovery, and this genus also contains members capable of degrading phenanthrene (29) and causing disease (24). Thus, many of the OTUs differentially abundant in CEWAF-driven dysbiosis are attributable to microbial groups that play a role in hydrocarbon degradation and/or fish disease. It will be important to identify if the specific bacterial strains in these groups are opportunistic pathogens and/or PAH degraders to understand the effects of these changes on fish health.

The microbiota of fish recovering from CEWAF exposure was generally restored to a Seawater Control-type microbiota following 21 days in clean seawater. Few studies monitor the external microbiota of fishes following perturbations. Tarnecki et al. (41) found that dysbiosis in the external microbiota of common snook (Centropomus undecimalis) caused by captive rearing was greatly remediated within 2 days of acclimation in the wild environment. Captive common snook exposed to copper sulfate did not recover a wild-type microbiota 2 years following chemical treatment; however, these bacterial community alterations did not cause noticeable detriments to fish health (53). Investigations on the effects of disease and antibiotics on the external microbiota of seabass (Dicentrarchus labrax) (54) indicate the external microbiota takes longer than a week to recover and may return to a healthy state in 3 weeks; however, differences in beta diversity measures and a single dominant taxon persisted. In our study, CEWAF-exposed red snapper maintained relatively high levels of PAHs in the liver following the 3-week recovery period. Despite this, the external microbiota was similar to that of Seawater Controls at the overall community structure level. A few differentially abundant OTUs remained, including Exposure/Recovery fish having lower abundances of OTUs within the *Marinobacter* and *Halomonas* genera and having higher abundances of OTUs within the *Proteobacteria* class. A similar experimental design in southern flounder (Paralichthys lethostigma) indicated a lasting effect of crude oil, leading to an alteration of oxidative homeostasis after a 3-week recovery (14). Chemical reactions due to reactive oxygen species can alter sulfur and nitrogen metabolism, thereby changing microbiota structure and leading to dysbiosis (55). Therefore, it is not unreasonable to hypothesize that concentrations of PAHs in recovering snapper cause lasting oxidative stress that contributes to slightly altered microbiota, even 3 weeks post-CEWAF exposure.

In conclusion, CEWAF exposure induced dysbiosis in the red snapper external microbiota, enriching OTUs within genera containing known hydrocarbon degraders and potential pathogens. IgM data from this study suggest that fish maintained in clean seawater may launch a more consistent immune response following challenge with a bacterial pathogen than if they are continually exposed to CEWAF, as IgM expression decreases over time during chronic CEWAF exposure. CEWAF exposure for as little as 7 days was able to induce lesion formation in red snapper. This data summation indicates that continued exposure to CEWAF causes dysbiosis concurrent with significant reduction of immunoglobulin expression.

## MATERIALS AND METHODS

### Exposure systems.

Juvenile red snapper were obtained from, and the exposure study carried out at, the University of Southern Mississippi’s Gulf Coast Research Laboratory (GCRL). Fish were exposed to the chemically enhanced water-accommodated fraction of Corexit 9500/DWH oil (CEWAF) and/or bacteria in a flowthrough system consisting of 24 tanks, each holding 75-L. Water was maintained at 20°C, 15-ppt salinity, dissolved oxygen content of >5 mg/L, and a pH of 8.5. Fish were maintained on a 16-h/8-h light/dark cycle and fed daily. Each tank held 4 fish, and each treatment was replicated in 4 tanks, for a total of 16 fish per treatment. This number was selected to allow for sampling of at least 6 individuals at two time points per treatment (*n* = 12) while providing additional individuals to account for potential mortalities. Fish were weighed in grams prior to stocking. There were six treatments, which included a control, a challenge/recovery treatment, two exposure/recovery treatments, and two chronic exposure treatments (Fig. 1): (i) Seawater Control, seawater for 28 days; (ii) Bacteria/Recovery, seawater for 7 days, a 1-h bacterial challenge, and then seawater for 21 days; (iii) CEWAF/Recovery, CEWAF for 7 days followed by clean seawater for 21 days; (iv) CEWAF/Bacteria/Recovery, CEWAF for 7 days, a 1-h pathogen challenge, and then seawater for 21 days; (v) CEWAF, CEWAF for 28 days; and (vi) CEWAF/Bacteria/CEWAF, CEWAF for 7 days, a 1-h pathogen challenge, and then CEWAF for 21 days.

During CEWAF exposure periods, fish were continuously exposed to Corexit 9500/DWH crude oil at a flow rate of 2 L/h. During periods of clean seawater exposure, fish were exposed to seawater at the same flow rate. The targeted nominal CEWAF exposure solution of 1 ppm total petroleum hydrocarbons (TPH), an environmentally relevant concentration found in Gulf of Mexico subsurface water samples collected during and after the DWH oil spill (56), was prepared fresh every 48 h following standard protocols issued by the Chemical Response to Oil Spills Ecological Effects Research Forum (CROSERF) (57). This stock CEWAF served as a source to supplement all exposure tanks at equal rates using a dilution system as in previous studies (12). Water quality (salinity, oxygen, pH, and temperature) and chemistry (ammonia, nitrite, nitrate, and alkalinity) were monitored and controlled to maintain appropriate environmental conditions during experimental trials. PAH concentrations were measured periodically in water of both exposure and control tanks, but liver PAH concentrations were used as the primary metric of PAH exposure. Bacterial challenges were performed with a Vibrio anguillarum strain (Listonella anguillarum [Bergeman] MacDonell and Colwell [ATCC 19264]), isolated from lesions in cod. This strain is of serotype O2, one of the dominant serotypes causing vibriosis in fish (25), for which Koch’s postulates have been demonstrated (https://www.atcc.org/products/19264). Fish were challenged with *V. anguillarum* at a concentration of 4.0 × 10^5^ CFU/mL of seawater. This concentration was chosen to reflect a slightly higher concentration than that used in previous research (13), as the previous study used juvenile flounder, which were smaller than the individuals used in the current study.

### Measurements and morphological observations.

During the exposure experiments, fish were observed for changes in behavior and morphology. All exposures were conducted under approved protocols: Mote Marine Laboratory’s Institutional Animal Care and Use Committee (IACUC; 18-04-KM2) for toxicity testing of oil and dispersed oil on marine fishes and the University of Southern Mississippi Coastal Sciences Department’s IACUC (19103001) for aquatic toxicological studies with small fish species. Sampling sizes are shown in Table 1. On day 14, 6 fish per treatment were randomly selected for sampling, with all 6 analyzed for liver PAH and 4 per treatment analyzed for immunoglobulin M (IgM) expression. The remaining fish were sampled on day 28 for liver PAH, with 4 randomly selected per treatment sampled for IgM expression and microbiota composition. Fish were euthanized and weighed in grams. These measurements took place following collection of microbiota swabs at day 28 to prevent potential contamination from the scale. Following measurements, tissues were dissected using aseptic techniques. Spleens for RNA analysis were placed immediately in RNAlater and stored at −20°C until analysis. Composite liver samples and tank exposure solutions collected for PAH analysis were stored in TraceClean glass jars at −20°C until analyzed.

### Water and liver PAH analysis.

Water samples (250 mL) were liquid-liquid extracted, and homogenized liver (2 to 5 g) was extracted using ASE 300 accelerated solvent extraction system by EPA method 3545. Due to the small size of the livers of fish used in this study, 2 individuals per treatment were randomly combined into a composite for PAH analysis. Prior to PAH extraction, samples were spiked with PAH surrogates naphthalene-d8, acenaphthene-d10, phenanthrene-d10, chrysene-d12, and perylene-d12. Surrogate recoveries were acceptable if they fell within the range of 70 to 120% recovery (EPA acceptance criteria). Liver samples were further purified by gel-permeation chromatography (GPC, SX3 Biobeads, 70 g in dichloromethane [DCM]) for lipid removal. Samples were then concentrated under nitrogen and exchanged with DCM for a final volume of 10 mL. PAH internal standards dibenzothiophene-d8 and benzo(e)pyrene-d12 were added before analysis. Extracts were analyzed by an Agilent 7890A gas chromatograph coupled to an Agilent 5975C mass selective detector. Analyte separation was achieved using a Zebron-5MS column (30 m by 0.250 mm by 0.250 μm) with helium as the carrier gas. Each sample batch was extracted with a method blank and laboratory control sample for quality assurance, and a continuing calibration verification was run at the beginning and end of every sequence and after every 10 samples. Gas chromatography-mass spectrometry selective ion monitoring (GC/MS-SIM) was used to analyze 48 PAHs following methods described by McDonald et al. (58).

### Immunoglobulin M expression.

IgM was measured from fish spleens using quantitative PCR. Total RNA was extracted from individual spleens using TRI reagent (Invitrogen) following the manufacturer’s protocol with the addition of a second final wash with 75% ethanol. Residual DNA was removed by treating the total RNA with Turbo DNase following the manufacturer’s protocol. One microgram of the DNase-treated RNA was reverse transcribed (RT) using the GoScript reverse transcription system (Promega) following the manufacturer’s protocol. Success of the RT was confirmed by endpoint PCR using the 18S housekeeping gene (HKG) and GoTaq green master mix (Promega), which included a no-RT control to test for DNA contamination and a no-template water control. The red snapper IgM and 18S gene-specific primers were obtained from Rodgers et al. (12). Quantitative PCR was performed using the PowerUp SYBR green master mix, in which 25 ng cDNA was combined with 300 nM (each) forward and reverse primer in a 15-μL reaction mixture. Samples were run in quadruplicate, including no-template blanks on a Chromo4 system (Bio-Rad) with a PCR cycle of 50°C for 2 min and 95°C for 2 min, followed by 44 cycles of 95°C for 15 s and 60°C for 60s. Relative expression was calculated using method described by Livak and Schmittgen (59), and IgM is reported as the log_2_-transformed threshold cycle (2^−ΔΔ^*^CT^*) calculated against the Seawater Control group.

### Microbiota characterization.

To determine long-term effects of exposures on the external microbiota, skin mucosal swabs were collected from four randomly selected red snapper per treatment on day 28. Sterile swabs (Puritan sterile cotton-tipped applicators, individually wrapped; Puritan Medical Products Company LLC, Guilford, ME) were coated thoroughly with mucus from the fish’s right side beneath the dorsal fin. Swabs were stored in sterile cryogenic vials and immediately placed in liquid nitrogen. DNA was extracted from swabs using the DNeasy PowerSoil kit (Qiagen, Valencia, CA) following the manufacturer's instructions. Sequencing of the 16S rRNA gene V4 hypervariable region was performed at MR DNA (Shallowater, TX [www.mrdnalab.com]) following established protocols. Briefly, primers 515F (5′-GTGCCAGCMGCCGCGGTAA-3′) and 806R (5′-GGACTACHVGGGTWTCTAAT-3′) with barcodes on the forward primer were used to amplify the V4 region using the HotStarTaq Plus master mix kit (Qiagen, Valencia, CA) as follows: 94°C for 3 min, then 30 cycles of 94°C for 30 s, 53°C for 40 s, and 72°C for 1 min, followed by a 5-min elongation at 72°C. PCR products were verified on a 2% agarose gel. Samples were pooled in equal proportions based on molecular weight and DNA concentration and purified using calibrated Ampure XP beads. Paired-end sequencing was performed on an Illumina MiSeq (2 × 300 bp) platform (Illumina, Inc., San Diego, CA).

Sequences were processed using the MiSeq standard operating procedure (SOP) (60), accessed 19 August 2019, in Mothur v1.42.3. Sequences less than 275 bp, containing homopolymer stretches greater than 8 bp, flagged as chimeras, or classified as *Archaea*, *Eukarya*, mitochondria, chloroplast, or unknown at the domain level were removed from the analysis. Sequence processing resulted in final sequences of approximately 252 bp. Operational taxonomic units (OTUs) were defined at 97% sequence similarity and classified using the Silva v132 database (61). Good’s coverage, rarefaction curves, and alpha diversity were determined following normalization to the sample with the fewest sequences (34,783 sequences), with species richness indicated by the total number of OTUs within a sample and species evenness calculated using the Shannon evenness index. Phylogenetic diversity and Simpson diversity indices were also calculated.

### Data analysis.

Alpha diversity measures were compared among treatments using ANOVA followed with Tukey’s *post hoc* tests. The microbiota community was analyzed via compositional data analysis (62, 63). OTU tables resulting from Mothur were loaded into R 4.0.3 as a phyloseq object using the phyloseq package (64). The microbiome package (65) was implemented to transform the data using the centered log ratio (CLR) transformation. Differentially abundant OTUs between treatments were determined using ALDEx2 (66) and ANCOM (67). CLR-transformed OTU tables were loaded into Primer v6 (68). Resemblance matrices were formed using Aitchison distance (62) and visualized using nonmetric multidimensional scaling (MDS) and principal-component analysis (PCA). Differences between communities were determined using PERMANOVA and ANOSIM with Primer v6. Taxa containing potential fish pathogens were designated according to the book by Austin and Austin (24), whereas those containing potential hydrocarbon degraders were chosen based on the reviews by Prince et al. (28) and McGenity (29). Potential hydrocarbon degraders were correlated with IgM expression and liver ∑^48^ PAH using partial least-squares (PLS) regression and visualized using clustered image maps (CIM) with the mixOmics package 6.14.0 (69) in R.

Relationships between treatment and physiological responses were determined using general linear models (GLM) using the GLM function in R 4.0.3 (70). Where appropriate, Tukey’s honestly significant difference tests were used to analyze multiple comparisons among treatments. All GLM distributions were assumed normal, as supported by data visualization (histograms and qq-plots) and model residuals, and no recommended transformations from Box-Cox analyses better fit model assumptions of normality and homogeneity of variances.

### Data availability.

Data are publicly available through the Gulf of Mexico Research Initiative Information & Data Cooperative (GRIIDC) at https://data.gulfresearchinitiative.org (https://doi.org/10.7266/n7-8804-yj09; https://doi.org/10.7266/n7-za4m-yx30; https://doi.org/10.7266/NE9XYA5C). The microbiota data sets generated during the current study are available in the Sequence Read Archive repository (www.ncbi.nlm.nih.gov/sra/) under SRA study accession no. SRP227352.
