# Supplementary material for: Dispersed Crude Oil Induces Dysbiosis in the Red Snapper Lutjanus campechanus External Microbiota
Source: Microbiol Spectr. 2022 Jan 26;10(1):e00587-21. doi: 10.1128/spectrum.00587-21 (PMC8791192; doi:10.1128/spectrum.00587-21)
Supplement: SUPPLEMENTAL FILE 2 — Supplemental material. Download SPECTRUM00587-21_Supp_2_seq10.pdf, PDF file, 2.8 MB [file spectrum00587-21_supp_2_seq10.pdf]

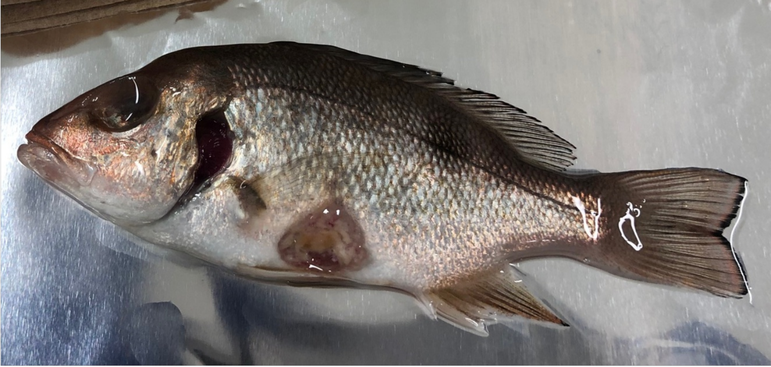

Figure S1. Image of red snapper from CEWAF/Bacteria/Recovery at Day 28 with an external lesion.

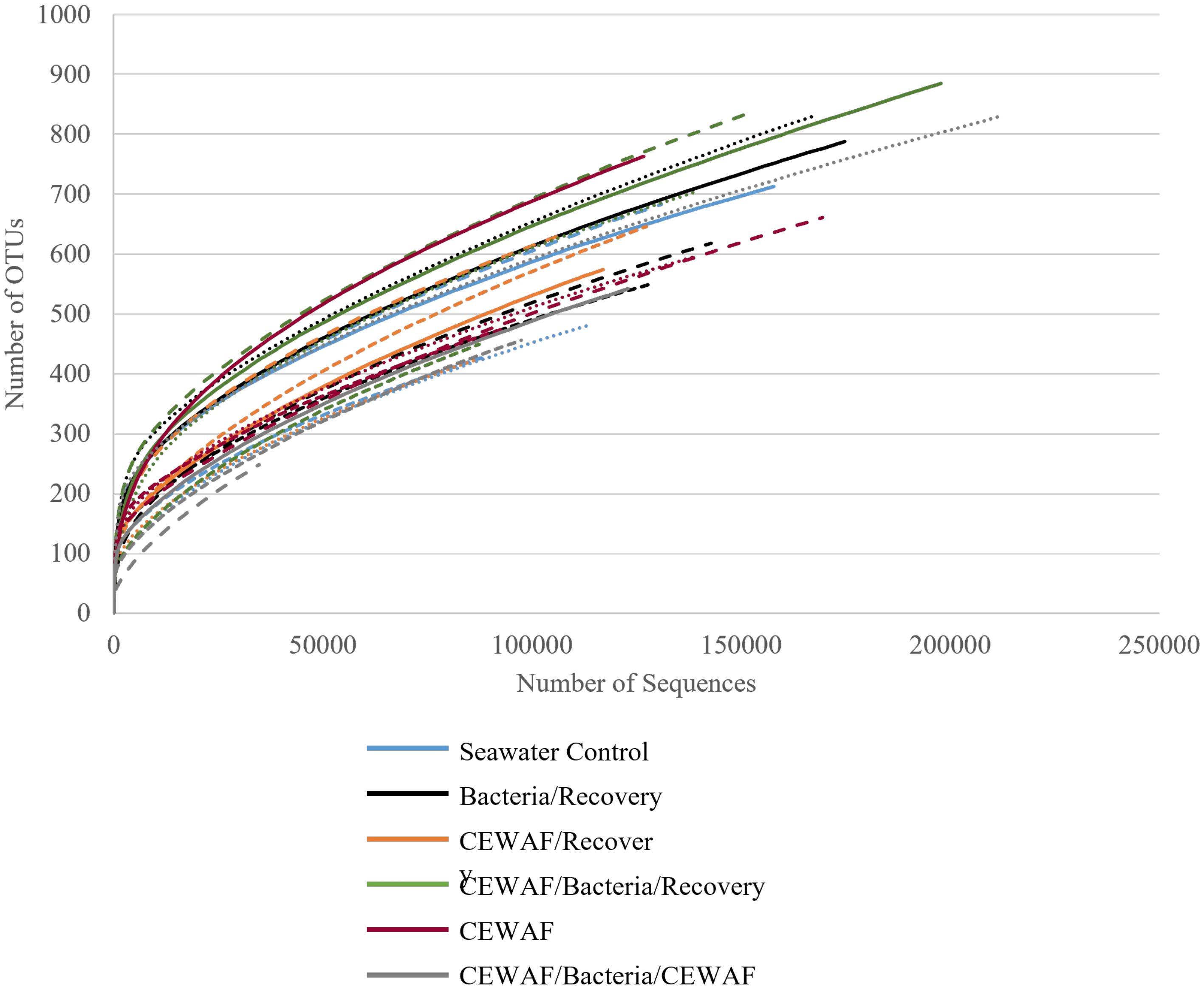

Figure S2. Rarefaction curves indicating sequence coverage of red snapper external microbiota. Individuals within each treatment are indicated with various dashed lines.

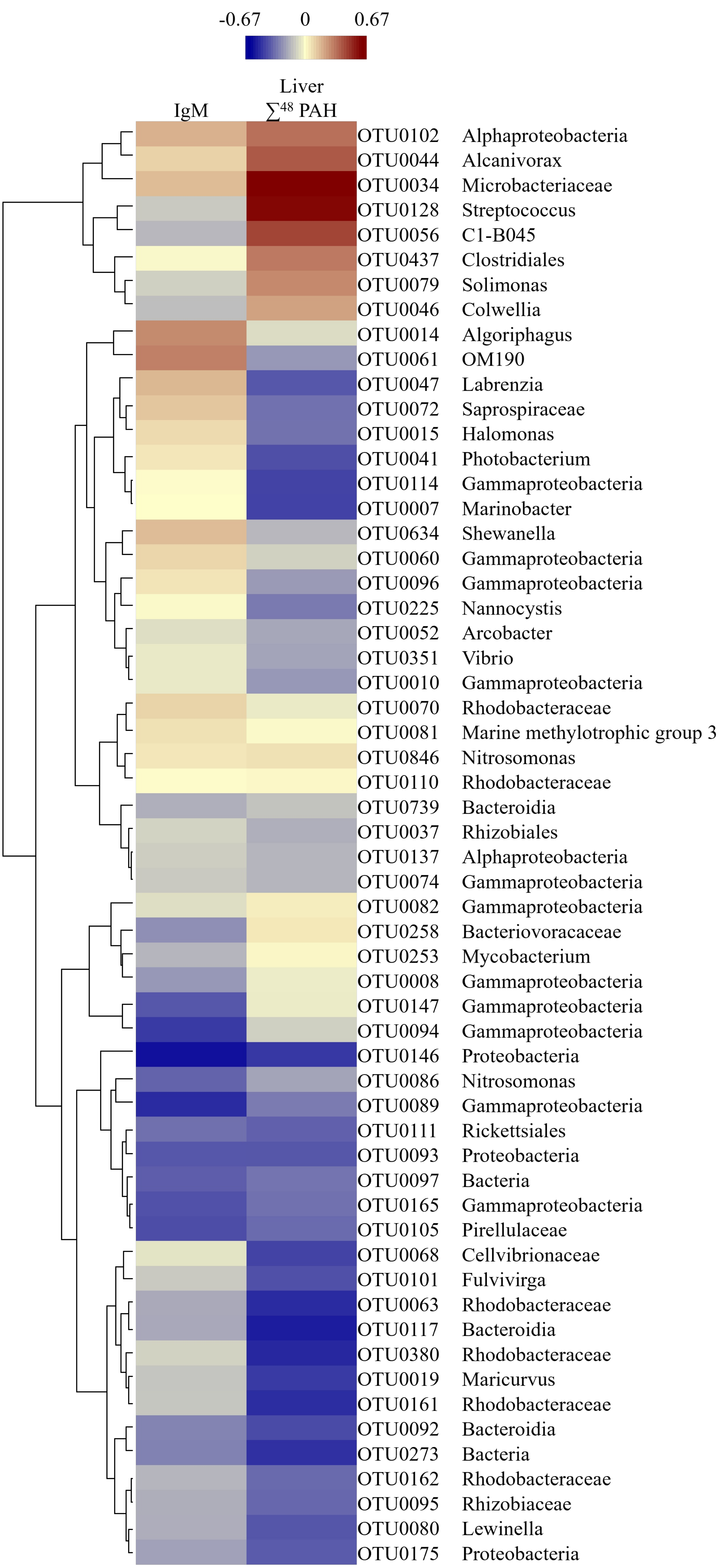

Figure S3. All correlations between OTUs and liver ∑^{48}PAH and IgM expression.

Table S1. Individual PAHs (ng/g ww) in the analyzed livers at Day 14.

	14 Day Sampling											
**PAH**	**Seawater Control**	**SD**	**Bacteria/ Recovery**	**SD**	**CEWAF**	**SD**	**CEWAF/ Bacteria/ CEWAF**	**SD**	**CEWAF/ Recovery**	**SD**	**CEWAF/ Bacteria/ Recovery**	**SD**
Naphthalene	0.00	0.00	52.45	24.49	85.33	39.59	94.85	33.26	123.87	52.73	99.73	41.17
C1N	4.05	7.02	66.20	21.28	191.01	79.59	243.12	125.47	110.69	54.67	116.48	40.61
C2N	0.00	0.00	98.43	38.59	446.96	160.48	543.02	236.28	246.00	90.61	209.51	50.00
C3N	0.00	0.00	85.30	35.46	288.70	138.90	309.74	139.58	194.39	97.77	168.52	49.95
C4N	0.00	0.00	24.66	26.29	114.37	86.03	121.73	64.70	33.32	30.65	89.78	79.47
Acenaphthylene	0.00	0.00	0.00	0.00	0.00	0.00	0.00	0.00	0.00	0.00	0.00	0.00
Acenaphthene	0.00	0.00	11.30	10.80	20.85	5.88	24.00	12.71	31.51	28.70	29.06	17.43
Fluorene	0.00	0.00	35.84	11.64	58.97	30.94	79.83	19.70	53.22	20.10	47.29	9.18
C1F	26.63	28.78	0.00	0.00	72.52	45.02	114.11	32.60	104.36	33.61	114.85	57.98
C2F	47.75	27.34	0.00	0.00	0.00	0.00	0.00	0.00	0.00	0.00	104.28	180.62
C3F	36.72	35.77	0.00	0.00	0.00	0.00	0.00	0.00	0.00	0.00	98.43	170.49
Dibenzothiophene	0.00	0.00	9.79	9.47	10.88	9.62	10.13	8.78	8.52	7.73	10.48	10.61
C1D	0.00	0.00	0.00	0.00	0.00	0.00	0.00	0.00	0.00	0.00	0.00	0.00
C2D	0.00	0.00	0.00	0.00	0.00	0.00	0.00	0.00	0.00	0.00	0.00	0.00
C3D	0.00	0.00	0.00	0.00	0.00	0.00	0.00	0.00	0.00	0.00	0.00	0.00
C4D	0.00	0.00	0.00	0.00	0.00	0.00	0.00	0.00	0.00	0.00	0.00	0.00
Phenanthrene	0.00	0.00	91.18	27.97	55.46	38.71	73.99	18.83	68.81	26.94	91.84	7.55
Anthracene	0.00	0.00	0.00	0.00	0.00	0.00	0.00	0.00	0.00	0.00	0.00	0.00
C1PH/A	0.00	0.00	80.42	39.36	46.05	29.65	81.80	42.31	114.71	49.00	102.75	13.62
C2PH/A	0.00	0.00	0.00	0.00	0.00	0.00	26.38	28.98	8.98	15.55	49.65	45.73
C3PH/A	0.00	0.00	0.00	0.00	0.00	0.00	14.43	25.00	6.13	10.62	0.00	0.00
C4PH/A	0.00	0.00	0.00	0.00	0.00	0.00	0.00	0.00	0.00	0.00	0.00	0.00
Fluoranthene	0.00	0.00	8.93	3.21	8.61	14.92	0.00	0.00	4.05	7.02	13.43	11.71
Pyrene	0.00	0.00	7.45	12.91	3.07	5.33	0.00	0.00	4.36	7.55	7.34	12.71
C1F/Py	0.00	0.00	0.00	0.00	0.00	0.00	0.00	0.00	0.00	0.00	0.00	0.00
C2F/Py	0.00	0.00	0.00	0.00	0.00	0.00	0.00	0.00	0.00	0.00	0.00	0.00
C3F/Py	0.00	0.00	0.00	0.00	0.00	0.00	0.00	0.00	0.00	0.00	0.00	0.00
C4F/Py	0.00	0.00	0.00	0.00	0.00	0.00	0.00	0.00	0.00	0.00	0.00	0.00
Benzo[B]fluorene	0.00	0.00	0.00	0.00	0.00	0.00	0.00	0.00	0.00	0.00	0.00	0.00

Napthobenzothiophene	0.00	0.00	0.00	0.00	3.40	5.89	0.00	0.00	0.00	0.00	9.52	16.48
C1NBT	0.00	0.00	0.00	0.00	0.00	0.00	0.00	0.00	0.00	0.00	0.00	0.00
C2NBT	0.00	0.00	0.00	0.00	0.00	0.00	0.00	0.00	0.00	0.00	0.00	0.00
C3NBT	0.00	0.00	0.00	0.00	0.00	0.00	0.00	0.00	0.00	0.00	0.00	0.00
C4NBT	0.00	0.00	0.00	0.00	0.00	0.00	0.00	0.00	0.00	0.00	0.00	0.00
Benzo[A]anthracene	0.00	0.00	0.00	0.00	0.00	0.00	0.00	0.00	0.00	0.00	0.00	0.00
Chrysene	0.00	0.00	0.00	0.00	0.00	0.00	0.00	0.00	0.00	0.00	0.00	0.00
C1BA/C	0.00	0.00	0.00	0.00	0.00	0.00	0.00	0.00	0.00	0.00	0.00	0.00
C2BA/C	0.00	0.00	0.00	0.00	0.00	0.00	0.00	0.00	0.00	0.00	0.00	0.00
C3BA/C	0.00	0.00	0.00	0.00	0.00	0.00	0.00	0.00	0.00	0.00	0.00	0.00
C4BA/C	0.00	0.00	0.00	0.00	0.00	0.00	0.00	0.00	0.00	0.00	0.00	0.00
Benzo[B]fluoranthene	0.00	0.00	0.00	0.00	0.00	0.00	0.00	0.00	0.00	0.00	0.00	0.00
Benzo[K]fluoranthene	0.00	0.00	0.00	0.00	0.00	0.00	0.00	0.00	0.00	0.00	0.00	0.00
Benzo[E]pyrene	0.00	0.00	0.00	0.00	0.00	0.00	0.00	0.00	0.00	0.00	3.32	5.75
Benzo[A]pyrene	0.00	0.00	0.00	0.00	0.00	0.00	0.00	0.00	0.00	0.00	0.00	0.00
Perylene	0.00	0.00	0.00	0.00	0.00	0.00	0.00	0.00	0.00	0.00	0.00	0.00
Indeno[1,2,3,-cd]pyrene	0.00	0.00	0.00	0.00	0.00	0.00	0.00	0.00	0.00	0.00	0.00	0.00
Dibenzo[a,h]anthracene	0.00	0.00	0.00	0.00	0.00	0.00	0.00	0.00	0.00	0.00	0.00	0.00
Benzo[g,h,i]perylene	0.00	0.00	0.00	0.00	0.00	0.00	0.00	0.00	0.00	0.00	0.00	0.00
**ΣPAHs (ng/g)**	**115.16**	**80.18**	**571.96**	**213.21**	**1406.19**	**610.39**	**1737.12**	**646.90**	**1112.91**	**422.41**	**1366.25**	**539.07**

Ave. Minimum detection limit 9.57 ng/g

Table S2. Individual PAHs (ng/g ww) in the analyzed livers at Day 28.

	28 Day Sampling											
**PAH**	**Seawater Control**	**SD**	**Bacteria/ Recovery**	**SD**	**CEWAF**	**SD**	**CEWAF/ Bacteria/CEWAF**	**SD**	**CEWAF/ Recovery**	**SD**	**CEWAF/ Bacteria/ Recovery**	**SD**
Naphthalene	0.00	0.00	36.82	32.94	202.90	119.46	115.12	29.29	33.72	1.85	70.80	59.65
C1N	4.68	8.10	81.13	24.65	767.16	455.36	538.99	218.87	73.38	19.05	307.39	283.88
C2N	6.69	11.58	100.86	9.96	1083.68	715.37	1263.52	290.50	88.09	54.43	211.86	98.06
C3N	6.44	11.16	81.77	34.94	562.57	423.76	909.96	202.03	67.29	38.42	181.89	100.32
C4N	0.00	0.00	11.67	23.35	237.83	153.11	382.59	230.41	7.00	0.00	31.18	54.00
Acenaphthylene	0.00	0.00	0.00	0.00	0.00	0.00	0.00	0.00	0.00	0.00	0.00	0.00
Acenaphthene	0.00	0.00	4.53	9.07	43.90	31.99	29.52	37.34	2.72	0.00	3.84	6.65
Fluorene	0.00	0.00	28.85	15.28	153.80	50.15	184.12	47.66	9.12	13.10	30.44	29.21
C1F	0.00	0.00	0.00	0.00	96.56	81.33	178.57	114.38	0.00	0.00	0.00	0.00
C2F	20.54	35.58	0.00	0.00	0.00	0.00	0.00	0.00	0.00	0.00	0.00	0.00
C3F	0.00	0.00	0.00	0.00	0.00	0.00	0.00	0.00	0.00	0.00	0.00	0.00
Dibenzothiophene	9.29	16.10	5.79	6.75	46.17	23.95	36.07	9.02	4.37	6.77	16.37	17.06
C1D	14.65	13.72	0.00	0.00	0.00	0.00	0.00	0.00	0.00	0.00	0.00	0.00
C2D	6.84	11.85	0.00	0.00	0.00	0.00	0.00	0.00	0.00	0.00	0.00	0.00
C3D	0.00	0.00	0.00	0.00	0.00	0.00	0.00	0.00	0.00	0.00	0.00	0.00
C4D	0.00	0.00	0.00	0.00	0.00	0.00	0.00	0.00	0.00	0.00	0.00	0.00
Phenanthrene	5.62	9.73	61.62	12.31	263.91	150.35	190.67	72.94	31.43	18.69	103.66	107.91
Anthracene	0.00	0.00	0.00	0.00	3.06	6.11	7.60	17.00	0.00	0.00	9.33	16.15
C1PH/A	7.08	12.26	17.80	35.60	231.01	111.83	198.90	69.08	55.59	66.62	143.92	156.86
C2PH/A	0.00	0.00	0.00	0.00	118.98	179.46	31.92	43.84	0.00	0.00	0.00	0.00
C3PH/A	0.00	0.00	0.00	0.00	0.00	0.00	0.00	0.00	0.00	0.00	0.00	0.00
C4PH/A	0.00	0.00	0.00	0.00	0.00	0.00	0.00	0.00	0.00	0.00	0.00	0.00
Fluoranthene	0.00	0.00	5.26	6.17	49.89	37.49	14.82	26.58	7.45	10.21	35.78	42.88
Pyrene	0.00	0.00	3.59	7.18	44.17	30.56	11.63	16.53	4.90	7.94	35.46	34.36
C1F/Py	0.00	0.00	0.00	0.00	0.00	0.00	5.93	13.26	11.33	32.72	23.67	41.00
C2F/Py	0.00	0.00	0.00	0.00	0.00	0.00	0.00	0.00	0.00	0.00	0.00	0.00
C3F/Py	0.00	0.00	0.00	0.00	0.00	0.00	0.00	0.00	0.00	0.00	0.00	0.00
C4F/Py	0.00	0.00	0.00	0.00	0.00	0.00	0.00	0.00	0.00	0.00	0.00	0.00
Benzo[B]fluorene	0.00	0.00	0.00	0.00	0.00	0.00	3.26	7.29	0.00	0.00	0.00	0.00

Napthobenzothiophene	0.00	0.00	0.00	0.00	5.89	11.78	2.08	4.65	0.00	0.00	0.00	0.00
C1NBT	0.00	0.00	0.00	0.00	0.00	0.00	0.00	0.00	0.00	0.00	0.00	0.00
C2NBT	0.00	0.00	0.00	0.00	0.00	0.00	0.00	0.00	0.00	0.00	0.00	0.00
C3NBT	0.00	0.00	0.00	0.00	0.00	0.00	0.00	0.00	0.00	0.00	0.00	0.00
C4NBT	0.00	0.00	0.00	0.00	0.00	0.00	0.00	0.00	0.00	0.00	0.00	0.00
Benzo[A]anthracene	0.00	0.00	0.00	0.00	9.34	18.68	5.73	12.81	0.00	0.00	9.24	16.01
Chrysene	0.00	0.00	0.00	0.00	8.59	17.17	3.63	8.12	0.00	0.00	10.61	18.38
C1BA/C	0.00	0.00	0.00	0.00	0.00	0.00	0.00	0.00	0.00	0.00	0.00	0.00
C2BA/C	0.00	0.00	0.00	0.00	0.00	0.00	0.00	0.00	0.00	0.00	0.00	0.00
C3BA/C	0.00	0.00	0.00	0.00	0.00	0.00	0.00	0.00	0.00	0.00	0.00	0.00
C4BA/C	0.00	0.00	0.00	0.00	0.00	0.00	0.00	0.00	0.00	0.00	0.00	0.00
Benzo[B]fluoranthene	0.00	0.00	0.00	0.00	0.00	0.00	0.00	0.00	0.00	0.00	0.00	0.00
Benzo[K]fluoranthene	0.00	0.00	0.00	0.00	0.00	0.00	2.38	5.32	0.00	0.00	0.00	0.00
Benzo[E]pyrene	0.00	0.00	0.00	0.00	4.95	9.90	0.00	0.00	0.00	0.00	0.00	0.00
Benzo[A]pyrene	0.00	0.00	0.00	0.00	2.73	5.47	2.88	6.43	0.00	0.00	0.00	0.00
Perylene	0.00	0.00	0.00	0.00	0.00	0.00	2.77	6.18	0.00	0.00	0.00	0.00
Indeno[1,2,3,-cd]pyrene	0.00	0.00	0.00	0.00	0.00	0.00	0.00	0.00	0.00	0.00	0.00	0.00
Dibenzo[a,h]anthracene	0.00	0.00	0.00	0.00	0.00	0.00	0.00	0.00	0.00	0.00	0.00	0.00
Benzo[g,h,i]perylene	0.00	0.00	0.00	0.00	0.00	0.00	0.00	0.00	0.00	0.00	0.00	0.00
**ΣPAHs (ng/g)**	**81.84**	**30.28**	**439.70**	**42.51**	**3937.09**	**2164.88**	**4122.66**	**736.29**	**551.57**	**205.57**	**1225.43**	**991.87**

Ave. Minimum detection limit 9.57 ng/g
